# Cultural adaptation of the Condom Use Self Efficacy Scale (CUSES) in Ghana

**DOI:** 10.1186/1471-2458-10-227

**Published:** 2010-04-30

**Authors:** Kwaku O Asante, Paul N Doku

**Affiliations:** 1 Regent University College of Science and Technology, Department of Human Development and Psychology, City Campus, Dansoman, Ghana; 2 Community Based Sciences, Section of Psychological Medicine, University of Glasgow, UK

## Abstract

**Background:**

Accurate assessment of self-reports of sexual behaviours is vital to the evaluation of HIV prevention and family planning interventions. This investigation was to determine the cross-cultural suitability of the Condom Use Self Efficacy Scale (CUSES) originally developed for American adolescents and young adults by examining the structure and psychometric properties.

**Method:**

A self-administered cross-sectional survey of a convenient sample of 511 participants from a private university in Ghana with mean age 21.59 years.

**Result:**

A Principal Component Analysis with varimax rotation identified a 14 item scale with four reliable factors labelled Appropriation (Cronbach alpha = .85), Assertive (Cronbach alpha = .90), Pleasure and Intoxicant (Cronbach alpha = .83), and STDs (Cronbach alpha = .81) that altogether explained 73.72% of the total variance. The scale correlated well with a measure of condom use at past sexual encounter (r = .73), indicating evidence of construct and discriminatory validity. The factor loadings were similar to the original CUSES scale but not identical suggesting relevant cultural variations.

**Conclusion:**

The 14 item scale (CUSES-G) is a reliable and valid instrument for assessing condom use self efficacy. It is culturally appropriate for use among Ghanaian youth to gauge actual condom use and to evaluate interventions meant to increase condom use. Finally, the study cautioned researchers against the use of the original CUSES without validation in African settings and contexts.

## Background

HIV/AIDS infections continue to be one of the world's greatest public health challenges as no vaccines have been found for curative treatment of the deadly disease. The HIV virus is mainly spread through sexual intercourse and the main hope to prevent infection remains modification of sexual behaviours including correct and consistent condom use [1]. Condoms offer safe, economically cheap and practically effective means of preventing both unwanted pregnancies and sexually transmitted infections including HIV/AIDS when used consistently and properly. This prevention strategy is however hindered by low use of condom especially by people living in areas with HIV/AIDS epidemic although condoms are readily available [2]. People's sexual behaviours take place in complex socio-cultural settings and motivations for condom use are complex and intricate involving a range of levels such as individual, couple and community [1]. These notwithstanding, there is urgent need to understand factors influencing condom use to inform designing of effective preventive strategies [2,3].

Availability of condoms is important but the evidence so far suggests that other factors must be at play for individuals to actually use them. Consistent condom use is linked to high levels of self efficacy [4]. Self efficacy emphasizes the individual and entails general influences that are "concerned not with the skills one has but with judgements of what one can do with whatever skills one possesses" [5]. Self efficacy reflects a person's level of confidence in his or her ability to control the environment [6]. A person whose cognitive self-evaluation or judgement of their capabilities is high is also able to influence all manner of his or her human experiences. However, a good predictor of ones's condom use intentions can only aid effective intervention programs if efficacy related to condom use is reliably and validly measured [7]. Thus, to further investigate self efficacy correlates of intended and actual condom use, a valid and reliable outcome is demanded. There is no such measure specifically developed for use in Africa. This calls for the development of a new scale or adaption and validation of an existing one. Adapting an existing one is both economically effective and practical and has the added advantage of permitting comparisons with data from other parts of the world. Several parameters have been specifically developed to evaluate condom use self efficacy and their impact on actual condom use with moderate success [8]. The Condom Use Self Efficacy Scale (CUSES) developed by Bradford and Beck represents one such valid and reliable instrument that is quick and easy to administer [4]. The CUSES originally developed for English speaking American population has wide acceptance and has been translated, adapted and used in several countries [4,9-14]. The CUSES which has 4 subscales and 13 more non-associated items offers a comprehensive approach to assess condom use self efficacy in new settings without much alteration, addition or removal of items from the scale.

Despite the wide use of the CUSES in studies conducted in Africa, the scale has not been validated [15-21]. Cross cultural adaptation and validation of the CUSES for Ghana would represent a major advance in the process of identifying condom use behaviours of young people in Ghana and would facilitate collaborative studies in comparing studies in Ghana with other countries. Therefore the aim of this study was to validate the psychometric properties of the CUSES in Ghana; analyse the factor structure of the questionnaire; and evaluate the predictive power (construct validity) of the CUSES among Ghanaian youth by comparing it with actual condom use among university students.

## Method

### Sample and participants

The sample in the study comprised 511 students of Regent University College of Science and Technology, a private university in the capital city of Ghana, Accra. The first researcher is a staff of the university and that explained the convenient nature of the sample chosen for the study. The sample size represents 98% of response rate from eligible prospective individuals approached to take part in the study.

### Measures

#### Demographic Data

Participants' personal characteristics such as age, sex, year in university, religious affiliation, place of residence (affluent area or otherwise), mother's educational level, ethnic background, marital status, sexual intercourse experience, and condom use.

#### Self efficacy

The Condom Uses Self Efficacy Scale (CUSES) was administered to the participants to assess condom use self efficacy [4,22,23]. The measure consists of items about individual's perceived ability to use condoms with a higher internal consistency (Cronbach alpha of .91) and a two-week test-retest reliability of .81. The CUSES uses a 5-point Likert scale that ranges from strongly agree to strongly disagree. Higher scores indicate stronger or higher perception of condom use efficacy, after reversing negatively worded items. The scale contains 28 items but a later analysis to find subscales left 13 items unassigned [9]. The original CUSES has four subscales: Mechanics (items 1, 27, 14, 22); Partner Disapproval (items 9, 10, 16, 17, 18); Assertive (items 4, 5, 6); Intoxicants (items 24, 25, 28) with internal consistencies of .78, .81, .80 and .82 respectively [9]. The total score on the 28 item scale ranges from 0 to 112.

### Procedure

A list of registered courses for the semester was collected from the university registry and the researchers contacted fellow university lecturers from different programs in the university and asked them to explain the research to the students and hand out the questionnaires. All lecturers contacted agreed except one who explained that the students would have to move just after the class to a different lecture immediately after her lesson and she is not prepared to offer the last 20 minutes of her lecture period for the purpose of this research. The participants were appropriately informed about the rationale of the survey and the voluntary nature of it, and they were appropriately assured of the anonymity and confidentiality of their answers. The students were informed not to indicate their names on the form. Finally, the students were asked if they had understood the instructions. The questionnaires were then distributed to all students in the class as none indicated otherwise. The researchers counted the number of questionnaires distributed. Thus the anonymous survey questionnaire was administered to the students in their lecture halls. These took place at the end of their classes, and students then filled in the questionnaires and left them on their desks which the researchers collected after all students were gone and checked them for completion. It took an average of 20 minutes to complete the questionnaire. The survey was filled out during a 1-h course. Participation was not required and students had the opportunity to refuse cooperation. In all, three students did not answer any item at all on the forms whilst two questionnaires were not left on the desk. Thus the number of non respondents was 5. The study was approved by both the Ghana Health Service Ethical Review Committee and Human Research Committee of Regent University College of Science & Technology.

### Data Analyses Strategy

The Statistical Package for Social Sciences (SPSS) version 16 software program was used for the data analyses. Descriptive analyses on Means, Standard Deviations, Frequencies and Percentages were initially performed as data cleaning technique. Then a Principal Component Analysis (PCA), varimux rotation, a useful statistical technique was applied on the data as the technique is common and relevant for findings patterns, hidden and simplified structures that often underlie data of high dimension such as the one in this study. In the analysis all the items on the CUSES were entered on an equal footing. In the first PCA conducted, 8 components were extracted with eigenvalues greater than 1 that would explain 75.02% of the total variance. The PCA designation criteria adopted required that Kaiser-Meyer-Olkin Measure of Sampling Adequacy (MSA) must be greater than 0.50 for each individual item as well as the set of items for their inclusion in a subsequent analysis. The overall MSA for the set of items included in the first analysis was 0.746 with a significant Bartlett test of Sphericity probability of < 0.001. However the MSA for some of the individual variables were less than 0.50 and were therefore excluded. In subsequent analyses, commonalities for variables that were less than 0.50 were also excluded from further analysis. Complex structures where one variable has high loadings or correlations (0.40 or greater) on more than one component were removed from the analysis. The step by step adherence to these designation criteria eliminated 14 items in the final PCA and extracted 4 factors. Internal consistencies for the scale were assessed by computing Cronbach's alpha and item-total correlation coefficients.

## Results

### Descriptive Data

The 511 participants that completed the scale comprised 54% males (n = 280) and 46% (n = 238) females with a mean age of 21.59 (SD = 4.74). Majority of the students (93.2%) were Christians and 74.3% were in the first year. Approximately 60% of the participants attended public senior secondary school and 59.5% live in affluent areas of Accra, Ghana's capital with their parents. Although 87.8% (n = 455) of the participants are single and never married, approximately 82% (n = 426) of them reported having been sexually active (have engaged in sexual intercourse). However, only 48% (n = 205) reported that they used condom during their last sexual encounter.

### Inferential Analysis

#### Construction of Ghana Version of the Condom Use Self Efficacy Scale (CUSES - G)

The PCA identified a 14 - item scale (CUSES-G) with high internal consistency (.91) that comprised of 4 main factors which we subsequently named "Appropriation", "Assertive", "Pleasure and Intoxicant", and "STDs" respectively. The total scale in the final analysis accounted for 73.72% of the total variance (Table 1) and has an MSA of 0.736 with a significant Bartlett test of Sphericity probability of < 0.001. Factor 1 accounted for 36.7% of the variance and consisted of five items with a high internal consistency (.85). Factor 2, with three items accounted for 15.3% of the variance. Factor 3 accounted for 11.6% of the variance and consisted of 3 items. The fourth and final factor accounted for 10.1% of the variance and consisted of 3 items. Cronbach alpha coefficient for factor 2, factor 3 and factor 4 were .90, and .83 .81 respectively. These internal consistencies are comparable to that of the original CUSES (Table 2). The factors also show between moderate to high inter-item correlation (Table 3). External validity of the extracted scale was evaluated by analyzing association between the scale, and condom use. Table 4 outlines these associations. Higher scores on each of the subscales (factors) as well as the total scale are positively correlated with higher condom use at last sexual encounter. A complementary independent t-test analysis on self efficacy between students who used condom at last sexual encounter and those who did not confirmed the associations reported in table 4. Students who used condom at last sexual encounter scored significantly higher on condom self efficacy than those who did not (t = 21.96, p < .001, Cohen d = 1.98) with an effect size correlation of 1.98.

**Table 1:** Factor Structure and Factor loading on the CUSES using Rotation Method: Varimax with Kaiser Normalization

	*Components*					
	
	M	SD	*1*	*2*	*3*	*4*
Percentage of Total Variance Explained			36.7	15.3	11.6	10.1
						
**FACTOR 1: APPROPRIATION**						
I would feel embarrassed to put a condom on myself or my partner	1.52	1.49	.683			
I feel confident I could gracefully remove and dispose of a condom when we have intercourse	1.52	1.24	.744			
I feel confident in my ability to incorporate putting a condom on myself or my partner into foreplay	1.77	1.31	685			
I feel confident that I could use a condom with a partner without "breaking the mood."	1.73	1.24	.809			
I feel confident that I could use a condom successfully	1.32	1.23	.820			
						
**FACTOR 2: ASSERTIVE**						
I feel confident in my ability to discuss condom usage with any partner I might have	1.21	1.24		.877		
I feel confident in my ability to suggest using condoms with a new partner	1.41	1.32		.888		
I feel confident I could suggest using a condom without my partner feeling "diseased"	1.67	1.28		.844		
						
**FACTOR 3: PLEASURE AND INTOXICANT**						
I feel confident I could use a condom during intercourse without reducing any sexual sensations	1.52	1.22			.726	
I feel confident that I would remember to use a condom even after I have been drinking	1.78	1.08			.863	
I feel confident that I would remember to use a condom even if I were high.	1.70	1.04			.900	
						
**FACTOR 4: STDs**						
I would not feel confident suggesting using condoms with a new partner because I would be afraid he or she would think I've had a homosexual experience	1.10	1.27				.671
I would not feel confident suggesting using condoms with a new partner because I would be afraid he or she would think I have a sexually transmitted disease	1.11	1.32				.934
I would not feel confident suggesting using condoms with a new partner because I would be afraid he or she would think I thought they had a sexually transmitted disease	1.10	1.27				.903

**Table 2:** Comparisons of factor structure internal consistencies of the present study (CUSES-G) and the Original CUSES Scale

Factors	Original CUSES		CUSES-G	
		
	Items	Alpha	Items	Alpha
Factor 1	4	.78	5	.85
Factor 2	5	.81	3	.90
Factor 3	3	.80	3	.83
Factor 4	3	.82	3	.81
Total Scale	15	.91	14	.93

**Table 3:** Inter-Item Correlation Matrix of the factors of the CUSES-G scale

Factors	Total Scale
Factor 1 (Appropriation)	.850
Factor 2 (Assertive)	.703
Factor 3 (Pleasure and Intoxicant)	.652
Factor 4 (STDs)	.506

**Table 4:** Correlation between the CUSES-G scale and condom use

Source	Appropriation	Assertive	Pleasure and Intoxicant	STDs	Total scale
Assertive (n = 511)	.463**				
Pleasure and Intoxicant (n = 511).	.436**	.358**			
STDs (n = 511)	.242**	.113*	112*		
Total scale (n = 511)	.850**	.703**	.652**	.506**	
Condom used at last sex (n = 426)	.527**	.550**	.387**	.544**	.730**

** Correlation is significant at the 0.01 level (2-tailed).

* Correlation is significant at the 0.05 level (2-tailed).

## Discussions

This study was undertaken to evaluate the appropriateness and practicability of the Condom Use Self Efficacy Scale (CUSES) among Ghanaian university students. Previous researches tested the scale against a sample of geographically diverse populations and found some cultural diversity; although the scale was found to have good reliability and construct validity [4,9,22,23]. This calls for cultural adaptation and validation of the scale before it becomes a sound instrument and a good predictor of condom use for populations different from the original American young people for which the scale was constructed. However, despite the use of the scale in several studies in Africa, the literature revealed that this is the first work designed to validate the factorial dimensions of the scale in the region.

Consistent with Bradford and Beck but with notable variations, the present study identified 4 factors (Appropriateness, Assertive, Pleasure and Intoxicants, and STDs) from the CUSES analysis that appears to be related to different dimensions of condom use self efficacy among Ghanaian youth [4]. However, the notable and relevant differences on particular items that loaded on each factor identified in the study reflect cultural differences related to condom use self efficacy, in Ghana implying that some items on the original scale are not relevant in the Ghanaian context.

The first factor which we called "Appropriateness" is more closely linked to the one labelled "Mechanics" by Brien et al. than the "Appropriation" labelled by Barkley and Burns [9,23]. Two items (I feel confident in my ability to put a condom on myself or my partner, and I feel confident in my ability to put a condom on myself or my partner quickly) well loaded in Brien et al. label were replaced by three other items (I would feel embarrassed to put a condom on myself or my partner, I feel confident in my ability to incorporate putting a condom on myself or my partner into foreplay, and I feel confident that I could use a condom with a partner without "breaking the mood") [9]. This factor dimension is indicative of self efficacy related to multifaceted appropriate condom use skills by self or a partner.

The second factor noted in this study is consistent with the "Assertive" label that Brien and colleagues found and shares the identical items [9]. We also called this factor "Assertive" as the loaded items are related to self efficacy dimensions of assertiveness, negotiation skills and ability to persuade a partner to use a condom. The emergence of this factor is indicative that Ghanaian adolescents and young adults, like their American counterparts, are becoming more confident in their ability to persuade, negotiate and discuss condom use with a partner, presumably because of increased awareness of risks associated with its non usage. Observing that the Ghanaian culture has a more conservative, religious and traditional beliefs about sexuality, marriage and condom use, the identification of this factor is encouraging. The third factor we identified, called "Pleasure and Intoxicants" is indicative of the ability to use condoms while under the influence of alcohol or drugs without feeling a reduction in sexual sensation. This factor is related to "Intoxicants" identified by Brien et al [9].

The final factor called "STDs" was the same as the one Barkley and Burn found [23]. Barkley and Burn (suggested that items on this factor involve fear (afraid) that may trigger physiological arousal suggesting that the factor represents the physiological feedback component of self efficacy [23]. The "STDs" is closely linked to "Partner Disapproval" that Brien et al. labelled except that two of their items (If I were to suggest using a condom to a partner, I would feel afraid that he or she would reject me and If I were unsure of my partner's feelings about using condoms, I would not suggest using one) could not have loads greater than 0.40 in our analysis [9]. STDs especially HIV/AIDS has gained prominence as a global health epidemic in recent years than say 20 years ago. All the items on the factor are related to stigma associated with STDs. Plausibly, this could underlie the emergence of STDs related items as a factor in the present study and that conducted by Barkley and Burn [23] but not in the case of Brien et al studies conducted 25 years ago [9,23].

Clearly, the changes in the composition of items that loaded on some of the factors identified in this study compared with earlier researches conducted in different countries indicate that cultural diversities are real when it comes to condom use self efficacy [9,12-14,23]. Observations in cultures such as Ghana, where premarital sex and condom use are regarded as a violation of religious, social and traditional norms, discussions of condom use are uncomfortable topics; issues that would affect one's self efficacy and condom use.

We examined the external validity of the factors by evaluating their association with actual condom use during the last sexual encounter. A bivariate correlation verified that the identified factors accurately represent condom use self efficacy and independently correlates positively with actual condom use. This finding was further supported by an independent t test analysis comparing those who used condom and those who did not on the CUSES -G. This demonstrates that perceived condom use self efficacy is an important predictor of condom use. Higher perceived condom use self efficacy seems to be related to control over sexual pleasure under the influence of alcohol and drugs, good appraisal of anticipated risks of STDs in sexual encounter situations, good assertiveness and enhanced negotiation skills.

## Conclusion

The most important finding of the present study is the observation of relevant cultural diversity similar to other previous studies [14,17-19], and [23]. This evidence suggests that the use of non-validated CUSES in other studies in African cultures could cause spurious findings. In this regard the paper urges' health agencies and researchers to use the 14 item CUSES-G in Ghana as the scale is culturally appropriate for use among Ghanaian youth to gauge actual condom use. The scale accounted for a reasonable proportion of the variance (73.72%), showed good internal consistency (.93) similar to those reported by earlier validation studies and had satisfactory construct validity as analyzed by condom use during the last sexual encounter [14,17-19,23]. The findings of the present investigation, however, should be interpreted with caution as the results are based on a convenience sample and the findings could not be generalized to the entire Ghanaian youth.

## Competing interests

The authors declare that they have no competing interests.

## Authors' contributions

Both authors contributed equally to the design, data collection and analysis. PND drafted the initial manuscript, and both authors read, edited and approved the final manuscript.

## Pre-publication history

The pre-publication history for this paper can be accessed here:

http://www.biomedcentral.com/1471-2458/10/227/prepub
